# Two-dimensional BN buffer for plasma enhanced atomic layer deposition of Al_2_O_3_ gate dielectrics on graphene field effect transistors

**DOI:** 10.1038/s41598-020-71108-5

**Published:** 2020-09-07

**Authors:** Michael Snure, Shivashankar R. Vangala, Timothy Prusnick, Gordon Grzybowski, Antonio Crespo, Kevin D. Leedy

**Affiliations:** 1 Air Force Research Laboratory, Sensors Directorate, Wright Patterson, AFB 45433 USA; 2 KBR, Beavercreek, OH 45433 USA

**Keywords:** Electronic devices, Electronic properties and devices

## Abstract

Here, we investigate the use of few-layer metal organic chemical vapor deposition (MOCVD) grown BN as a two-dimensional buffer layer for plasma enhanced atomic layer deposition (PE-ALD) of Al_2_O_3_ on graphene for top gated field effect transistors (FETs). The reactive nature of PE-ALD enables deposition of thin (2 nm) dielectrics directly on graphene and other two-dimensional materials without the need for a seed or functionalization layer; however, this also leads to significant oxidation of the graphene layer as observed by Raman. In FETs, we find this oxidation destroys conductivity in the graphene channel. By transferring thin (1.6 nm) MOCVD BN layers on top of graphene channels prior to PE-ALD, the graphene is protected from oxidation enabling BN/Al_2_O_3_ layers as thin as 4 nm. Raman and X-ray photoelectron spectroscopy on BN films show no significant oxidation caused by PE-ALD of Al_2_O_3_. Inserting the BN layer creates an atomically abrupt interface significantly reducing interface charges between the graphene and Al_2_O_3_ as compared to use of a 2 nm Al buffer layer. This results in a much smaller Dirac voltage (− 1 V) and hysteresis (0.9 V) when compared to FETs with the Al layer (V_Dirac_ = − 6.1 V and hysteresis = 2.9 V).

## Introduction

Devices based on two-dimensional (2D) materials offer great possibilities for applications requiring mechanical flexibility^1^, ultra-low power^2^, heterogeneous integration^3^, and extreme scaling^4^. The layered structure of these materials, with strong in-plane bonding and weak van der Waals inter-planar bonding, provides the ability to thin and stabilize them down to a single layer. Although, these materials have fully compensated surfaces with no dangling bonds; they are quite sensitive to the surrounding environment, including the substrate, ambient adsorbates, and in devices, the addition of component layers like-contacts, gate dielectrics, and passivation layers^5–10^. These can lead to oxidation, doping, and charge scattering severely degrading properties. In graphene, the effects of these surrounding elements have been shown to greatly impact basic transport properties and devices performance. To mitigate the effects of the substrate and adsorbates, a thin van der Waals (vdW) buffer layer, like hBN^11,12^, has shown to be extremely effective at preserving the properties of graphene.

The progression toward high performance 2D devices necessitates the integration with high quality thin dielectrics, for gates, tunneling barriers, etc.^13–15^; for which, atomic layer deposition (ALD) is ideally suited. However, 2D materials present a significant challenge to depositing ultra-thin, high quality, high-κ dielectrics due to the inherent lack of reactive surface nucleation sites^16^. To overcome these nucleation challenges, various surface functionalization and seeding layers have been used to promote ALD deposition. Surface functionalization by treatment with reactive species like ozone^17^, XeF_2_^18^, and H_2_, O_2_, or N_2_ plasma^19–21^ produce functional surface groups and defects that provide nucleation sites. However, these functionalization methods partially convert sp^2^ C bonds in graphene to sp^3^ degrading properties, which can be in some cases recovered through annealing^19^. Nucleation can also be improved by functionalization with physisorbed molecules like H_2_O^22^ and NO_2_^23^ followed by low temperature thermal ALD. Zheng et al.^22^ found the low temperature Al_2_O_3_ ALD layer to be low density and have low dielectric constant requiring deposition of a second higher temperature high quality layer. On the other hand, seeding-layers consisting of thin polymers^24^, metal-oxides^25,26^, or metals^27^ can be deposited by low impact methods, like evaporation, that minimize damage to the graphene layer. Functionalizing the graphene surface or inserting a seed layer between graphene and the ALD dielectric can introduce interfacial charges and traps, reduce the total dielectric constant of the gate stack, and limit the minimum equivalent oxide thickness (EOT)^4,22,28^. One alternative is to use a 2D buffer layer on top of the graphene to protect the interface during ALD^29,30^.

In this paper, we investigate the use of 2D BN grown by metal organic chemical vapor deposition (MOCVD) as a thin buffer layer for plasma enhanced (PE) ALD of Al_2_O_3_ on graphene for top gated field effect transistors (FETs). PE-ALD offers a number of advantages over thermal ALD for deposition of dielectrics on 2D materials. Generally, the high reactivity of the plasma species allows for a wider range of materials to be produced, with higher quality, higher density, and at lower temperatures than thermal ALD^31^. Plasma species can also modify the surface promoting nucleation and greatly reducing nucleation delay critical for reducing gate thickness^32^. Additionally, PE-ALD enables more exotic coating and high-*k* dielectric that can reduce (EOT) even further^33^. For graphene and other 2D materials the plasma species can serve as in-situ functionalization eliminating the need for ex-situ functionalization or seeding required for thermal ALD of ultra-thin high quality layers; however, this occurs at the cost of damaging the 2D layer^29,34^. By transferring a thin (1.6 nm) BN layer on top of the graphene FET channel prior to PE-ALD of the Al_2_O_3_ gate dielectric, we demonstrate a weakly interacting 2D buffer layer that protects the graphene layer during PE-ALD. We find that depositing directly on graphene with no seed layer causes significant oxidation destroying the graphene channel, while devices with a BN buffer layer are preserved. These BN devices also have significantly lower shift in the Dirac point and lower hysteresis due to reduced interfacial doping at the BN/graphene vdW interface.

## Results

**Figure 1:**
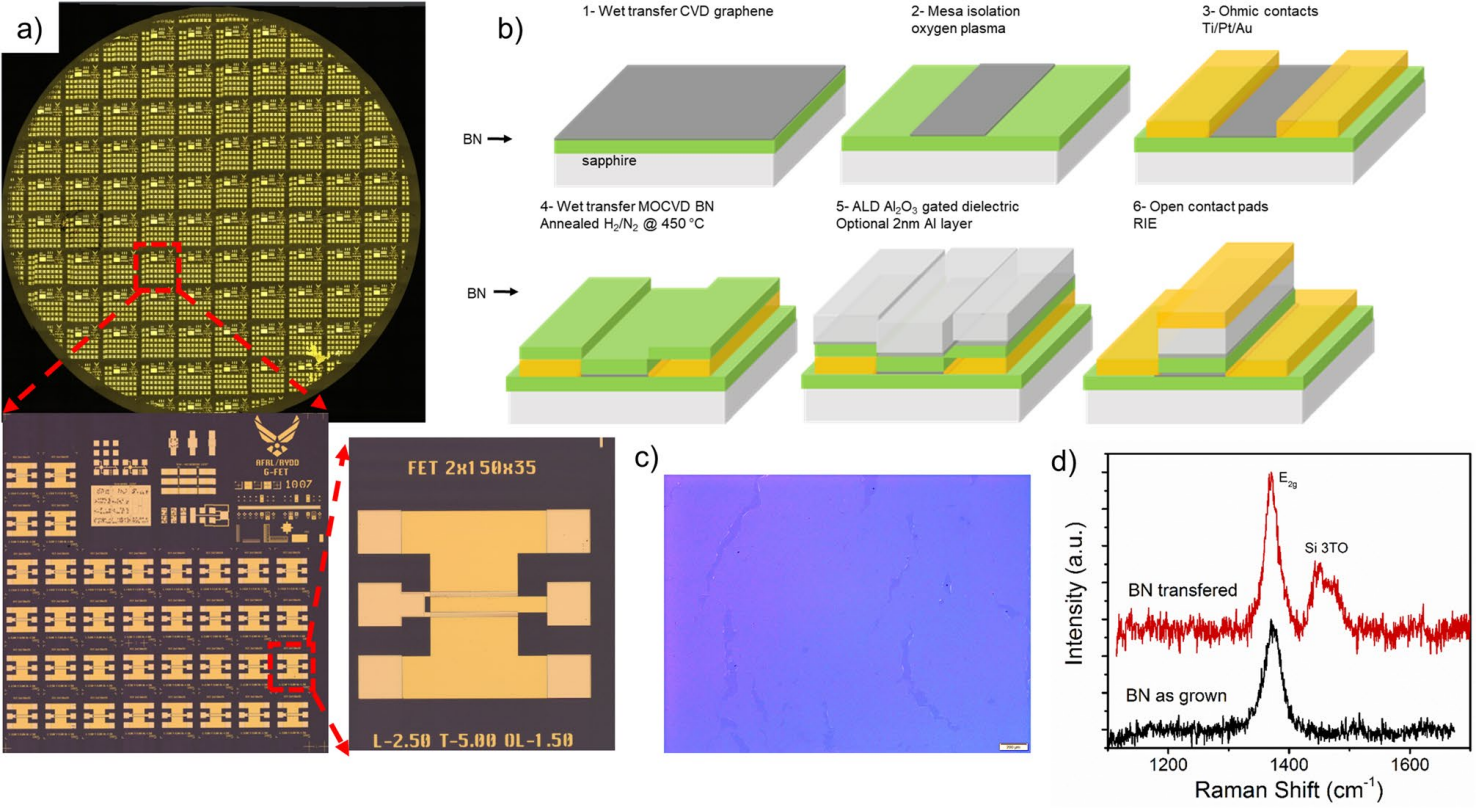
Graphene FET fabrication process. (**a**) Optical images of a full 2″ wafer, reticle, and single FET. (**b**) Process flow for device fabrication. (**c**) Optical image of MOCVD BN layer transferred to SiO2/Si and (**d**) Raman spectra of BN before and after transfer. The full 2″ wafer in (**a**) is a stitched image from multiple 5× images created using MetaMorph imaging software ver. 7.10 (www.moleculardevices.com).

Top-gated graphene FETs were fabricated on chemical vapor deposition (CVD) graphene films transferred to 2″ MOCVD grown BN on sapphire substrates. A variety of FETs with gate lengths from 2.5 to 10 µm were used to investigate electrical characteristics of devices with different buffer layers for PE-ALD Al_2_O_3_ gate dielectric, Fig. 1a. For this investigation, devices with four different buffer layers prior to deposition of 20 nm Al_2_O_3_ were explored: (1) no seed layer—PE-ALD directly on graphene, (2) transferred 2D BN on graphene followed by PE-ALD, (3) 2 nm e-beam evaporated Al seed layer followed by PE-ALD, and (4) transferred 2D BN layer followed by 2 nm e-beam evaporated Al seed layer and PE-ALD. Figure 1b shows the process flow for device fabrication. In this process, graphene mesas were defined by O_2_-plasma etching followed by e-beam deposition of Ti/Pt/Au ohmic contacts. An O_2_ plasma power of 200 W was used to selectively remove the graphene, while leaving the BN layer intact. For devices with the 2D BN layer a 1.6 nm thick BN layer was wet transferred on top of the defined graphene channel and contacts. BN layers were grown on 2″ sapphire wafers by MOCVD under self-terminating growth conductions, similar to the BN layer used on the substrates side, providing a large-area, highly reproducible and uniform 1.6 nm thick layer^35,36^. Figure 1c,d shows an optical image and Raman spectra from representative BN layers transferred to SiO_2_/Si. After, the BN is transferred, the PMMA is removed, and the stack annealed to improve the graphene BN interface and clean the BN surface prior to ALD. Next, a 2 nm Al seed layer, excluded in buffer layer 1 and 2, is deposited followed by 20 nm of Al_2_O_3_ by PE-ALD and Ti/Au gate contacts.

**Figure 2:**
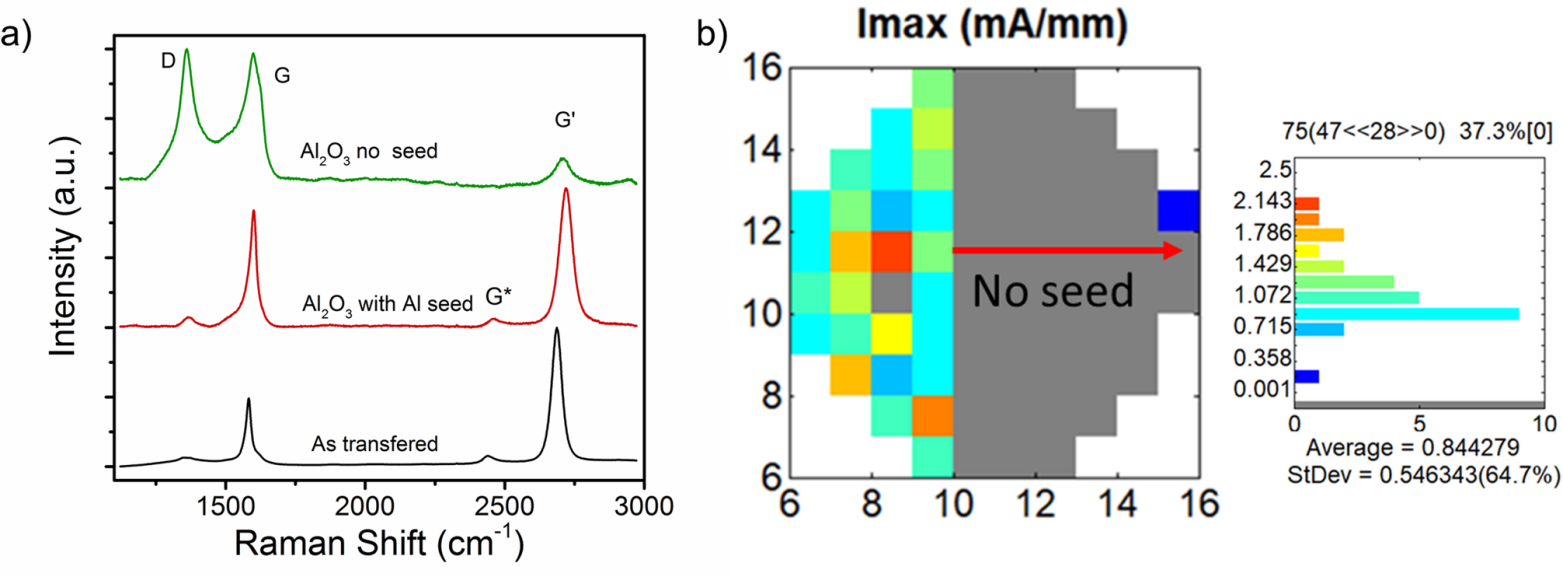
(**a**) Graphene Raman spectra comparing before and after PE-ALD of Al_2_O_3_ with Al seed and with no seed. (**b**) Map of maximum Ids from graphene FET wafer comparing effect of Al seed layer prior to Al_2_O_3_ deposition.

The advantages of PE-ALDs in-situ auto functionalization comes at the consequence of potentially damaging the graphene layer. Raman spectroscopy was used to investigate the effect of Al_2_O_3_ deposition on graphene’s structural quality. Figure 2a shows Raman spectra from a graphene layer transferred to sapphire before and after Al_2_O_3_ deposition. To compare the effect of the 2 nm Al layer, the graphene film was masked and Al was deposited on half of the sample. Compared to the as transferred spectra, the half without the Al layer shows a significant increase in defect related D/D’ peaks and reduction in the G’ indicative of the formation of graphene oxide^37^. The side with the 2 nm Al layer does not have the same increase in D and D’ indicating the layer serves to protect the graphene. AFM images (Fig. S1) of Al_2_O_3_ surface deposited directly on graphene and using an Al seed layers show a similar roughness (~ 1.3 nm RMS) and morphology. Both layers appear to completely cover the graphene layer with no signs of nucleation issues, which is expected due to the in-situ functionalization caused by the O_2_ plasma.

To investigate the effect on device operation, a full (2 × 150 µm) graphene FET wafer was processed, in ~ half of the devices the Al_2_O_3_ gate dielectrics were deposited with no seed layer and the other half with the Al seed layer. Device characteristics were measured on one device with a 10 µm gate length per reticle. Figure 2b shows a map of the maximum measured current from 95 devices across the wafer. The devices with no seed layer had no measured working devices out of the 57 measured, while the half with the Al seed layer had a yield of 95% working devices with a maximum I_D_ in the range of 0.7–2.1 mA/mm at V_ds_ of 0.1 V. The oxidation observed in Raman clearly has a significant effect on the electrical conductivity of the graphene layer.

**Figure 3:**
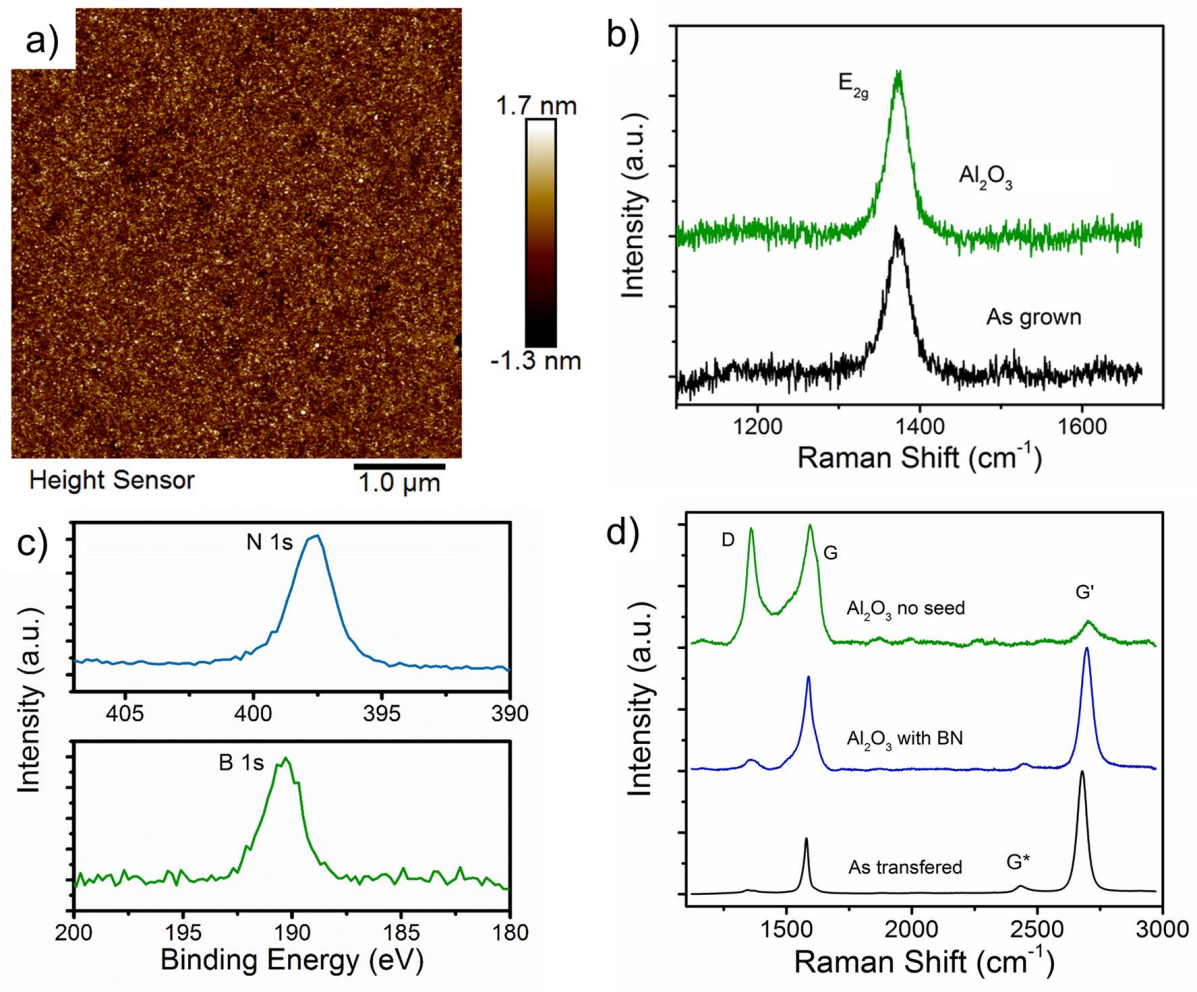
(**a**) AFM image of Al_2_O_3_ surface on BN/sapphire substrate. (**b**) Raman spectra from BN layer before and after Al_2_O_3_ deposition. (**c**) XPS spectra of N 1*s* and B 1*s* from BN layer under 2 nm of Al_2_O_3_. (**d**) Raman spectra comparing graphene before and after Al_2_O_3_ deposition with no seed layer and with the BN layer.

Although use of the Al layer preserves the graphene by protecting it from oxidation, it has been shown to introduce interface charges and traps causing unintentional doping and hysteresis in FETs^28,35^. To mitigate these interface effects we explore inserting a few layer thick sp^2^ bonded BN layer between the graphene and Al_2_O_3_ layer. Figure 3a–c show Raman, AFM and XPS from the as grown BN layer on sapphire before and after PE-ALD of Al_2_O_3_. BN is well established as an oxidation resistant coating even when thinned to only a few monolayers^38^, but how MOCVD/CVD grown films react to O_2_ plasma during PE-ALD is not established. AFM shows a uniform surface morphology with a roughness of ~ 0.5 nm (Fig. 3a). Comparison of before and after Raman shows little change in the E_2g_ mode of BN indicating no significant degradation to the BN structure. Investigation of oxidation of these BN films at high temperatures in O_2_ and high humidity, by Raman and XPS, show them to be stable above 800 °C (Fig. S2–S3). XPS analysis of the BN layer after PE-ALD of 2 nm Al_2_O_3_ shows only one photoelectron peak corresponding to the B 1 s (190.5 eV) and N 1 s ( 397.8 eV) in good agreement with previous reports on sp^2^ bonded BN^39^ with no oxide related peaks around 193 eV (B–O)^40^. The lack of oxidation signatures in the BN layer indicates the BN layer could be thinned to below four mono-layers. With the established stability of the BN layers under the PE-ALD process, we now investigate its use as a buffer layer for protecting graphene. Figure 3d shows the graphene Raman spectra from before and after PE-ALD Al_2_O_3_ on bare graphene and graphene covered with the BN buffer. As observed in Fig. 2, direct PE-ALD of Al_2_O_3_ oxidizes the graphene layer, but by covering the graphene with a thin BN layer the graphene is preserved with little change in the Raman. This can even be observed optically (Fig. S4) where the unprotected portion of graphene becomes completely transparent.

**Figure 4:**
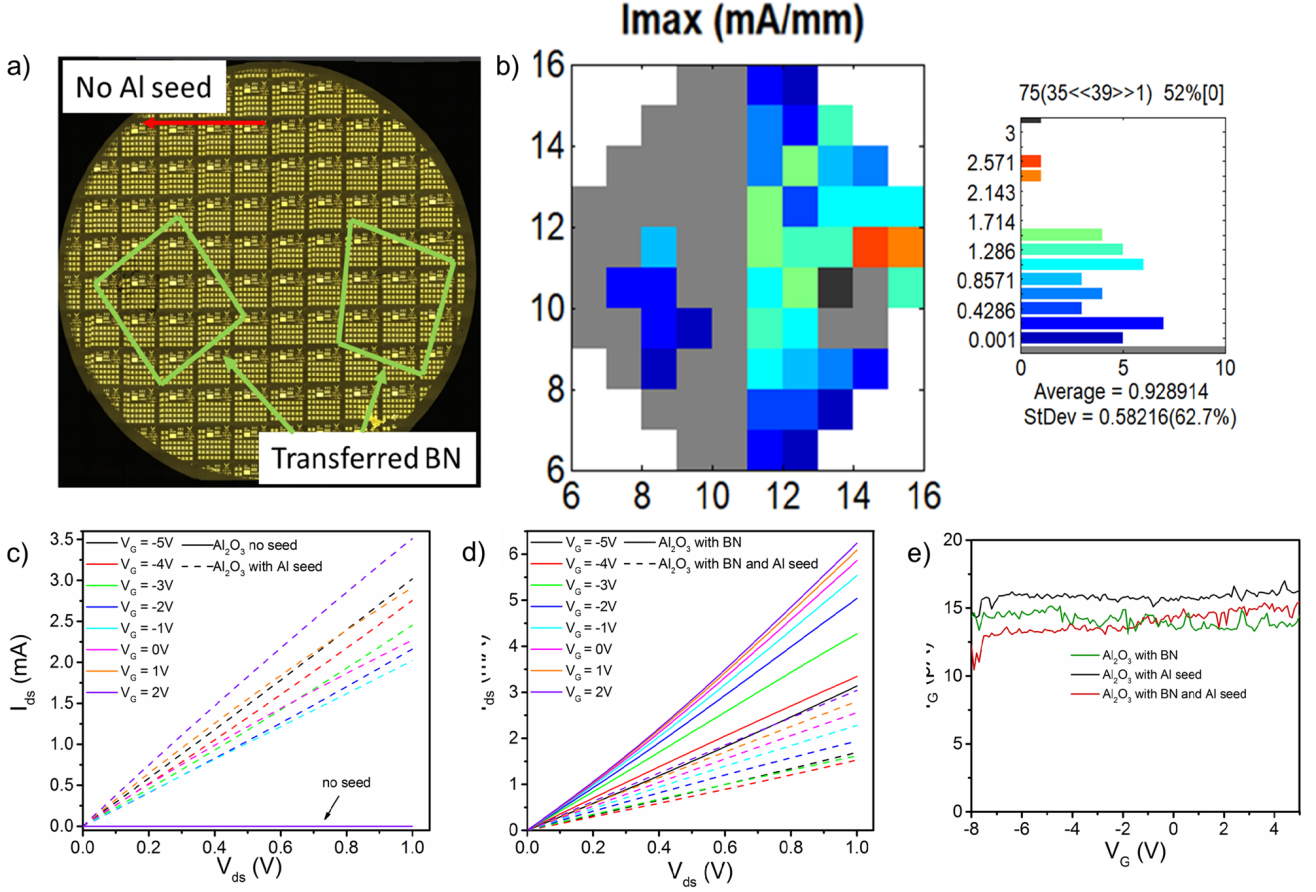
Graphene FETs comparing each of the 4 buffer types. (**a**) Optical image of the 2″ FET wafer indicating regions where BN was transferred (green rectangles) and Al seed deposited. (**b**) Map of maximum I_ds_ from the FET wafer. (**c**) Output curves from 10 µm gate length FETs with no seed and the BN layer and (**d**) with Al seed layer and with BN and Al seed layers. (**e**) Gate leakage current measured for the different buffer layer types at Vds = 0.1 V. The full 2″ wafer in (**a**) is a stitched image from multiple 5× images created using MetaMorph imaging software ver. 7.10 (www.moleculardevices.com).

To compare the effect of the 4 buffer types on FET performance, we fabricated a full wafer with top gates fabricated using each type, Fig. 4a. After mesas were defined and ohmic contacts deposited, two films of BN, from the same original wafer, were transferred on each half of the wafer, as denoted by the green rectangles in Fig. 4a. Then the Al layer was deposited on half of the wafer, similar to the wafer in Fig. 2, followed by PE-ALD of Al_2_O_3_. Wafers were mapped by I_ds_—V_G_ transfer curves from one transistor with a 10 µm gate length per reticle. Figure 4b shows the map of maximum measured current for each of these devices at V_ds_ = 0.1 V. Just as in Fig. 2 we see a clear delineation in operating devices corresponding to the use of the Al seed layer. However, here we see a cluster of functioning devices on the side with no Al layer where the BN film was transferred. Similarly, on the Al seeded side we see multiple functioning devices in regions where the BN was transferred. Figure 4c,d shows representative I_ds_–V_ds_ output curves from devices fabricated using each of the 4 buffer types. In the devices with the Al seed, a positive increase in I_ds_ with V_G_ from − 5 to 5 V was measured indicating majority electron carriers. Hall effect measurements from as-transferred graphene on BN/sapphire substrates found all samples were p-type prior to device processing with an average hole concentration of 4 × 10^12^ cm^-2^ and mobility of 1,860 cm^2^/Vs. This shift in carrier type after device fabrication is due to significant n-type doping caused by deposition of the Al_2_O_3_ gate dielectric^9,35^. Devices with only the BN buffer layer and both the BN and Al layer show a switch in the direction of I_ds_ dependence on V_G_. Hall effect measurements taken from van der Pauws (vdP) structures from each region (Fig. 1a top center of the recital) confirm this n-type doping with measured n_s_ (average of four vdPs) from vdPs with the BN buffer, Al seed layer, and both the BN and Al seed layer of − 9 × 10^11^, − 3 × 10^12^, and − 7 × 10^12^ cm^-2^ and Hall mobility of 890, 560, 1,060 cm^2^/Vs. To check that the buffer type does not impact the gate leakage current (I_G_), we measure I_G_ as V_G_ is swept from − 8 to 4 V at V_ds_ of 0.5 V. Figure 4e shows a very low I_G_ for all gate types, between 12 and 16 pA, demonstrating consistent insulating behavior of the gate dielectric independent of buffer type.

**Figure 5:**
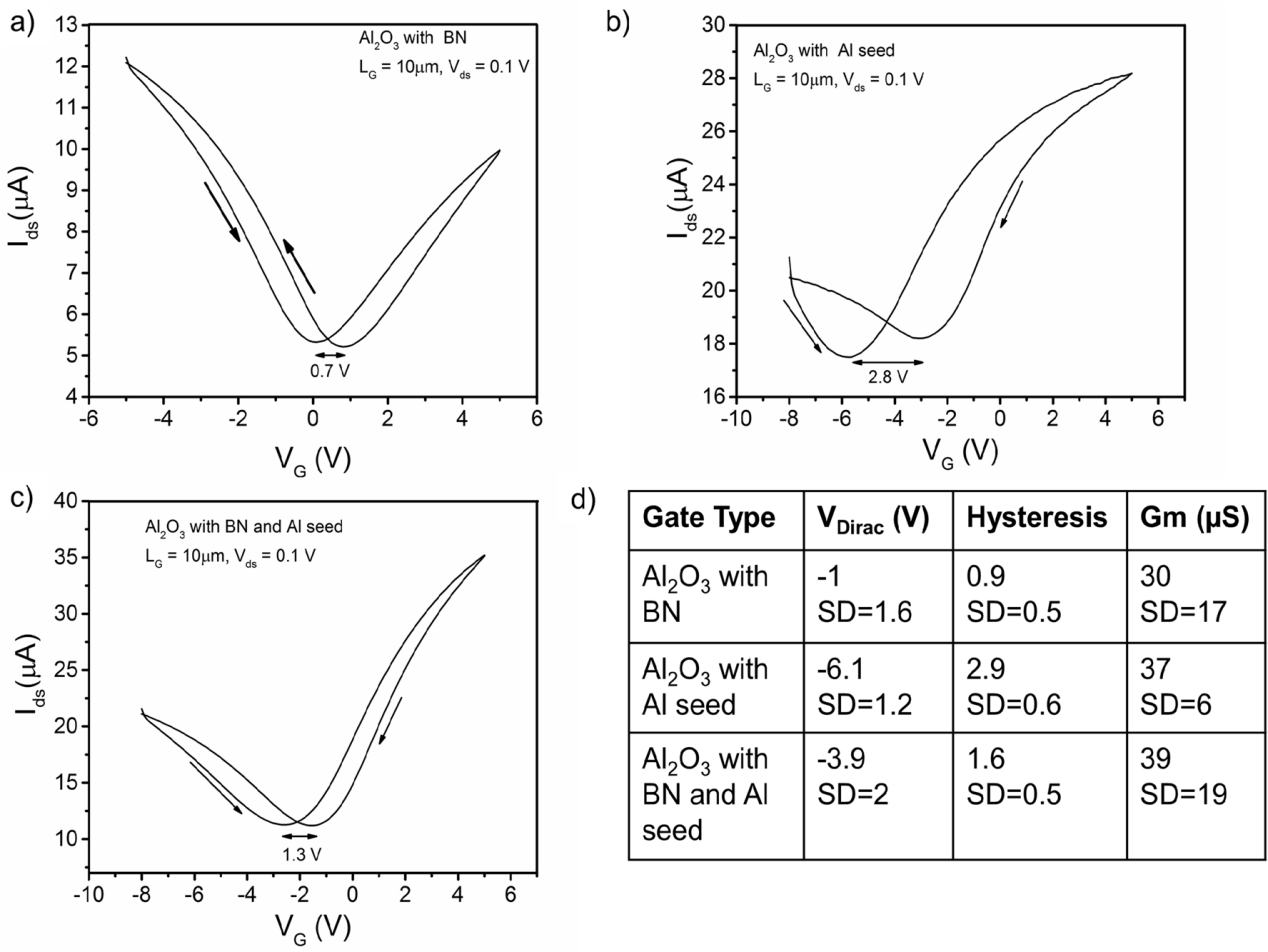
Transfer characteristics of graphene FETs with different buffer types (**a**–**c**). (**d**) Table summarizing mean V_Dirac_, hysteresis, and peak transconductance from at least 10 devices with each gate buffer type (std. = standard deviation).

Transfer curves for 10 µm gate length devices for each gate type are shown in Fig. 5. All device types show typical ambipolar behavior with a negative V_Dirac_ due to n-type doping and similar peak transconductance of 30 µS or better. Upon the reverse V_G_ sweep, hysteresis with a positive shift in V_Dirac_ is observed in I_ds_–V_G_, which is common in graphene FETs. This shift is due to negative charge traps at the graphene interface from defects in the dielectric and/or contamination (H_2_O, hydroxides, hydrocarbons)^41–43^. The effect of interface contamination can be reduced by processing under vacuum or inert environments, cleaning processes, annealing, or by inserting a 2D buffer layer, like BN. Indeed, if we compare transfer curves from each buffer type, we observe V_Dirac_ and hysteresis depend strongly on the buffer type with the lowest doping and hysteresis in devices with a BN buffer (Fig. 5d). This shows the inert van der Waals surface of sp^2^ BN forms a clean interface and effectively screens charges and doping from Al_2_O_3_^35^. Furthermore, if we compare devices with only the BN buffer layer and both the BN and Al buffer layers we observe a further reduction in doping and hysteresis by eliminating the Al layer. This Al layer, which oxidizes during exposure to air and O_2_ plasma during PE-ALD, is expected to be more highly defective having a higher concentration of traps than the PE-ALD Al_2_O_3_.

## Conclusion

We have reported on the use of few-layer MOCVD grown BN as an oxidation resistant buffer for PE-ALD deposition of Al_2_O_3_ of graphene FETs. The reactive nature of PE-ALD provides a path for deposition of the continuous Al_2_O_3_ dielectrics as thin as 2 nm without the need for a nucleation layer but causes oxidation of the graphene channel. Various buffer schemes were investigated to prevent oxidation of the graphene and improve device performance. Both a thin Al layer and transferred BN were shown to protect the graphene from significant oxidation and produce good device performance. The deposition of the Al_2_O_3_ dielectric contributed to a significant n-type doping causing a negative shift in the V_Dirac_ and hysteresis due to interface contamination and defects in the dielectric. Inserting a thin BN layer enabled reduction of these effects by providing a clean 2D dielectric interface with graphene and eliminating the need for the defective Al buffer layer. This work demonstrates significant improvements in graphene top gated FETs by using MOCVD grown 2D BN as a dielectric buffer, which is broadly applicable to other 2D materials prone to oxidation.

## Methods

Boron Nitride on 2″ sapphire substrates and transferred films used in this study were grown by metalorganic chemical vapor deposition (MOCVD) at 1,000 °C, 20 Torr and B/N ratio of 2250 for 30 min^36^. To transfer films, a 300 nm thick layer of PMMA was spun on the BN surface and cured at 180 °C. The BN layer was released by etching in a buffered oxide etch solution (BOE) at 20 °C for up to 2hrs. Released films were wet transferred, dried in air, followed by the PMMA removal with acetone, and finally annealed in 5% H_2_:N_2_ forming gas at 400 °C for 30 min. Graphene grown on copper by CVD using methane as the carbon source were transferred to the BN/sapphire substrates using a typical wet transfer with PMMA, similar to the process described in Ref.^44^.

The surface morphologies of graphene and BN layers were analyzed by AFM using a Bruker Dimension Icon in tapping mode. Raman measurements of graphene and BN were performed using a Renishaw inVia system under a backscattering geometry. A 488 nm excitation source at 4 mW, 20 µm slits, and a 3,000 l/mm grating was used for these measurements. Chemical analysis of films was performed using X-ray photoelectron spectroscopy (XPS, PerkinElmer Phi 5,500).

Graphene FETs were fabricated using transferred CVD graphene on BN/sapphire. The graphene mesas were etched using an O_2_-plasma. Source and drain Ti/Pt/Au contacts were evaporated. A 20-nm thick Al_2_O_3_ top gate dielectric was deposited by ALD. ALD was performed at 250 °C with half cycles of trimethylaluminum and a remote 300 W oxygen plasma, achieving a growth rate of 0.94 A/cycle. Gate metal (Ti/Au) contacts were defined using a second lift-off process^31^. The automated electronic characterization was performed using a Cascade Summit 12 k probe station with an HP 4142 parameter analyzer and GSG probes.

## Supplementary Information

Supplementary Information.
