# Supplementary material for: Two-dimensional BN buffer for plasma enhanced atomic layer deposition of Al2O3 gate dielectrics on graphene field effect transistors
Source: Sci Rep. 2020 Sep 7;10:14699. doi: 10.1038/s41598-020-71108-5 (PMC7477096; doi:10.1038/s41598-020-71108-5)
Supplement: Supplementary file 1 — Supplementary Information. [file 41598_2020_71108_MOESM1_ESM.pdf]

Two-dimensional BN buffer for plasma enhanced atomic layer deposition of Al_2O_3 gate dielectrics on graphene field effect transistors

Michael Snure[1,*], Shivashankar R. Vangala[1], Timothy Prusnick[2], Gordon Grzybowski[2], Antonio Crespo[1], Kevin Leedy[1]

[1]Air Force Research Laboratory, Sensors Directorate, Wright Patterson AFB, 45433, USA

[2]KBR, Beavercreek, OH 45433

* E-mail: michael.snure.1@us.af.mil, Tel: +1-973-713-8929

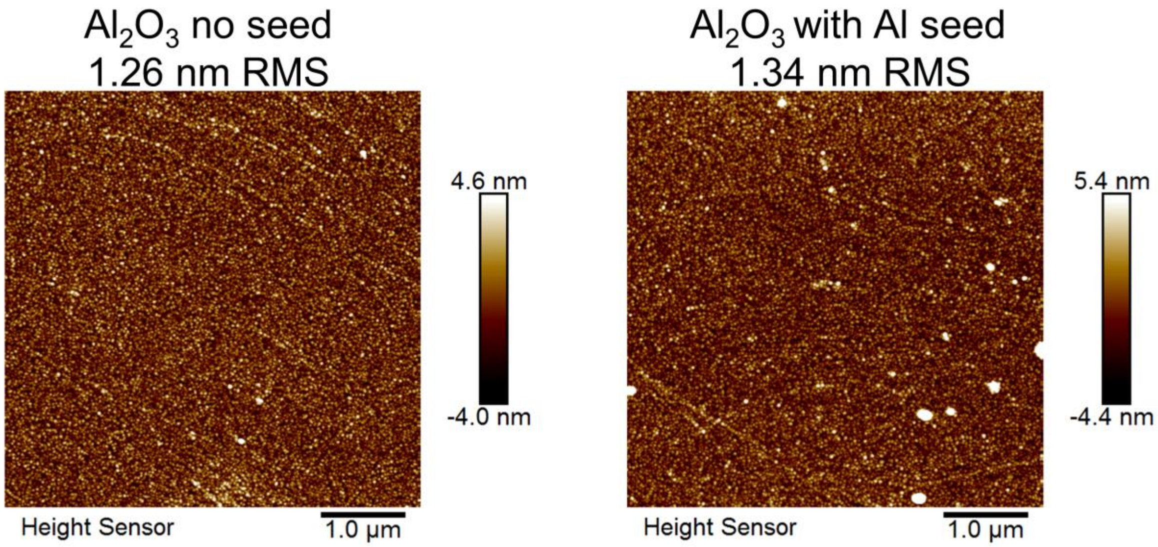

Figure S1: AFM images of Al_2O_3 surface on graphene with no seed and Al seed.

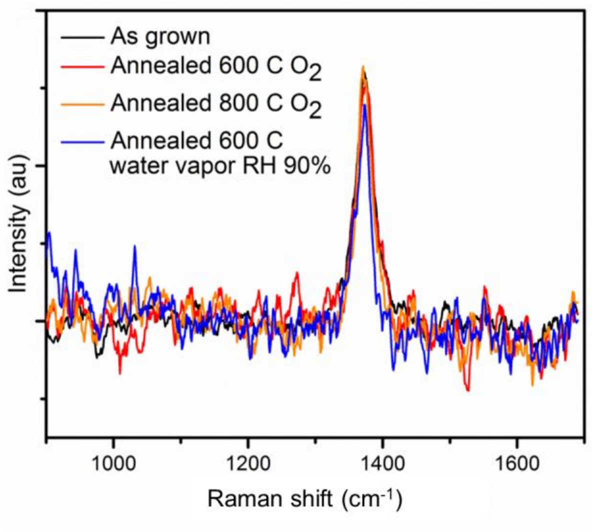

Figure S2: Raman spectra from BN layer on sapphire as grown, after annealing in pure O_2, and at a relative humidity of ~ 90%.

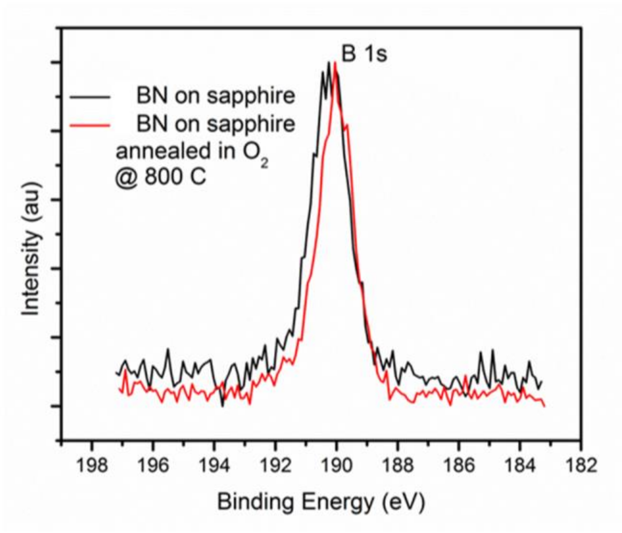

Figure S3: XPS spectra of B 1s from BN layer as grown and after annealing in O_2.

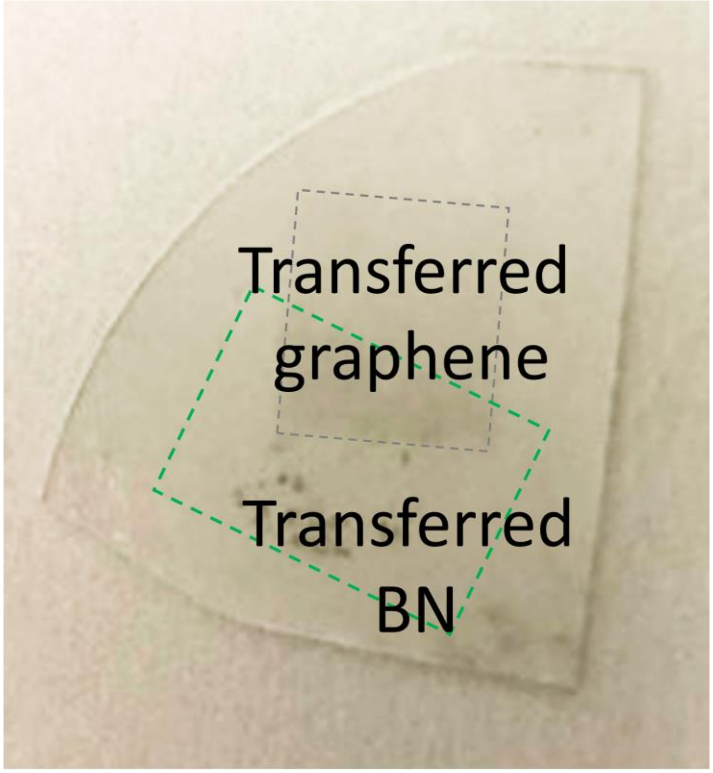

Figure S4: Optical image after PE-ALD of 20 nm Al_2O_3 on transferred graphene layer on BN/sapphire substrate. Half of the graphene layer was covered by 1.6 nm transferred BN layer prior to PE-ALD.
